# Intensive care and the different meanings of vulnerability

**DOI:** 10.5935/2965-2774.20230317-en

**Published:** 2023

**Authors:** Rebeca Mamede da Silva Alves, Rafael Casali Ribeiro

**Affiliations:** 1 Curso de Medicina, Faculdade de Odontologia de Bauru, Universidade de São Paulo - Bauru (SP), Brazil; 2 Centro Cuidar, Faculdade de Odontologia de Bauru, Universidade de São Paulo - Bauru (SP), Brazil

## INTRODUCTION

In the eyes of laypeople, intensive care may seem like a precise and objective field of study. Even to health professionals, believing that medical practice within intensive care units (ICUs) should be predominantly guided by technical decisions seems sensible and reasonable, even though there are nuances and some space for subjectivity. However, a careful look at particularities of the decision-making process in intensive care shows how different concepts and values, sometimes implicitly adopted, affect the ways intensivists think and, consequently, act. This article discusses a concept that largely intersects with the work processes in intensive care but remains poorly discussed: vulnerability.

### The different meanings of vulnerability

The term “vulnerability” is frequently used in medical literature, but bears multiple possible meanings that are commonly not explained. Usually, vulnerability indicates an inability to protect one’s own interests, susceptibility to damage and social determinants;^(1,2,3)^ in other words, vulnerability comprises internal (inability to protect) and external (social determinants) factors. In such a conception, it is understood as an attribute inherent to individual beings, in the same way it is used in most discussions of the subject seen on literature, which relates vulnerability to intensive care largely from a perspective of humanization or bioethics, and focuses on access to health care. However, understanding vulnerability as an individual experience misses its intersubjective and systemic dimensions, which engender the phenomenon, and thus limits possibilities of reflection and its reach on routine practices in specialized spaces such as the ICU. In such sense, vulnerability can serve as a type of safe conduct for social control and paternalism when interacting with critically ill patients, especially those who are objectively incapable of expressing themselves.^(1)^

In contrast to the first meaning, other concepts of vulnerability have developed out of more critical, dynamic and reconstructive perspectives. As Ayres^(4)^ explains, the concept of vulnerability develops and transforms in North American literature since its emergence in the 1990s around the AIDS epidemic, when vulnerability was initially understood according to the natural history of disease model described by Leavell and Clark, related to greater susceptibility to disease and poorer access to protection resources.^(5)^ At the time, the concept was structured following an ethical and rights guaranteeing perspective. Fundamentally, Brazilian literature on intensive care today still refers back to such a field of study.

After the onset of the AIDS epidemic, health-disease-care processes were resignified. Previously characterized as a linear relationship between aggressor and disease, the process is now understood as a result of mutual interactions amongst agents of disease, hosts and the environment, without there being an aggressor itself. Therefore, any agent (physical, chemical, or biological) becomes an aggressor due to physical, cognitive, affective and behavioral specificities of the host, which depend on the socioenvironmental context and the set of health knowledge and practices at their disposal.^(5)^ Thus, the natural history of disease model, when understood in its historicity, assumes that both the way in which a disease arises and develops and the ways in which the process itself is understood and interpreted determine the health-disease-care process.^(5)^

Such a conceptual transformation is reflected in Brazilian literature, especially in collective health studies, a critical and transdisciplinary field of knowledge resulting from the historical articulation between academia, health workers, management workers and social movements, inscribed in the movement known as Sanitary Reform.^(6)^ In this socially emancipatory perspective, vulnerability should not be understood only as a product of inequalities in power, material means or knowledge, to be compensated by decisions from health professionals, but also as a framework for examining and modifying health actions, that take into account social relationships, being them race, sex, class or other.

The discussion makes it clear how important it is not only to critically examine technical and scientific instruments used to intervene on people’s health, but also to criticize theoretical, conceptual and epistemological instruments, considering they also entail different and often inadvertent ethical, political and social consequences.^(4)^

### Intensive care

Caring for critically ill patients and emphasizing technical-scientific knowledge are particular features of ICUs.^(6)^ These highly specialized centers provide care using expensive technology to preserve life and restore the health of people whose condition is so critical that, without intervention, the risk of death is imminent.

In Brazil, where chronic underfunding of the Unified Health System (SUS - *Sistema Único de Saúde*) and other basic infrastructure needs to ensure living and health conditions for the majority of the population are not resolved,^(6)^ not only inequity in access to intensive care services is essential, but one must also pay attention to important limitations in access to other levels of care in the health system, in addition to broader health determinants that also affect access.^(6)^ Preventable diseases worsen and consequently require more intensive and painful treatments in a cascade of harmful effects, generating demand that is difficult to meet. Arguably, the problem requires systemic responses and is left to health professionals, above all, to master techniques required to provide critical care; and in that setting, although it is important to be aware of vulnerabilities, directly addressing them is not the health professionals responsibility. That is the path predominantly observed in health literature today: identifying vulnerability soon transitions into guaranteeing access to health services and guaranteeing patient autonomy.

In fact, access and autonomy are relevant issues, seeing as in ICUs patients are often unable to speak as a result of procedures (mechanical ventilation, sedation), lose the ability to control themselves and, at that moment, health professionals must serve as decision makers. However, the problem is not limited to this type of access because in such a scenario, health professionals must decide what is considered good for the patient. Notably, defining what is “good” is neither obvious nor easy and often involves ethical and political dilemmas.^(6)^ It is in that position that lies the importance of expanding the concept of vulnerability in intensive care – the position of deciding on behalf of others, in its multiple senses.

“Deciding on behalf of others” can be understood, in its most immediate meaning, as a process of unilaterally making decisions that affect the lives and, especially, the health of others; in this specific case, ICU patients. Another definition would be to make decisions *for* others, not through the health professional’s own techniques. Such a meaning will be developed later.

Deciding on behalf of others is obvious in cases where patients cannot communicate in the ICU, but, in such scenarios, even those who have the ability to decide - who speak, ask, deny, complain - still face limited autonomy.^(6)^ Often, patients in intensive care are not even given all relevant information to decide about their own care, and health professionals continue to choose for others. When the phenomenon falls within the scope of Brazilian public health, however, the issue becomes more complex. In Brazil, the other is often what literature calls “vulnerable”. The “vulnerable”, when in serious condition as are intensive care patients, is relegated to a state of double deprivation of autonomy: social vulnerability, which habitually places him or her in a situation of power disparity with the physician, which belongs to privileged social strata; and their critical health condition, admittedly dependent on decisions arising from the specialized knowledge of medical professionals.

### The intersection between vulnerability, technology and medical knowledge

Returning to Ayres’ discourse on the concept of vulnerability,^(4)^ the author pleads there are no inherently vulnerable individuals – as presented in the introduction – but rather people affected by different vulnerabilities, which come to life relationally and contextually. In other words, vulnerability is not an attribute of a person, but of a certain situation (historical, social, biological, geographical, political) that affects people unequally.

In such a conjunction, an individualizing concept, in which vulnerability is understood as a state of susceptibility and inability to protect oneself, implies that decisions rest with professionals and should revolve around ensuring access to necessary procedures. In dramatic scenarios such as intensive care, access to procedures that guarantee life are at stake, so repercussions of transferring the power to decide what is and is not “good”, from patients to health professionals, are overlooked. It is worth exploring what type of transformation in intensive care would arise from adopting a dialogic conception of vulnerability.

To address the issue we reference Merhy’s literature on health technologies. The author figuratively appropriates the image of a “suitcase” - a type of satchel or handbag, such as those used by physicians in the 19th and 20th centuries to store clinical instruments - to discuss different types of technology used by professionals during doctor‒patient encounters, which configure intersection processes.^(7)^ The author distinguishes three types of suitcases, technological toolboxes, knowledge and material and nonmaterial consequences, that a clinician makes use of in the care process: hard, soft-hard and soft technologies. Soft technologies are understood as those that mobilize relational resources between patients and professionals to produce care; soft-hard technologies mobilize professional knowledge, whether clinical, theoretical-conceptual, or epidemiological; and hard technologies are technological resources and materials that produce care, for example, a stethoscope, tomograph, mechanical ventilator and monitor.^(7)^

Modes of health care structured around predominantly soft technologies are therefore organized as relational spaces in which medical work is not fully captured by technological knowledge but competes with the user, forming a space of permanent dispute in which the production of care is unique.^(7)^ This type of technology prevails in the context of primary care, which is less supported by technological density and refers to the author’s own tenet that “there is no one way to perform clinical acts”. The presence of hard technology in the space is of lesser importance, and therapeutic encounters do not depend on technology.^(7)^ In contrast, modes structured around hard technologies are organized around equipment and, ultimately, the technical knowledge of health professionals, rarely utilizing the soft technologies suitcase and shifting the health model axis to the competence of physicians’ actions and procedures, which become punctual and subspecialized, sometimes practically nullifying properly said caregiving practices.^(7)^ It is within that space in which intensive care is usually located.

The field of soft technologies, inhabited by intersubjectivity, is more easily associated with concepts such as vulnerability and care. In contrast, it seems rational to consider that in intensive care units, one is justifiably abstracted from life complexities (relationships, territory, roles and autonomy), due to the imminent risk of biological bodily failure, and placed in a scenario of hard technological complexity that lasts until the organism no longer needs it. In this hypothetical scenario, there seems to be no conflict between the will of health professionals and patients. However, to Merhy, even technological medicine is permeated by a constitutive tension when producing health care, that is, the dispute between what professionals and service users want, because producing health care is a living act that seeks to conform health actions to certain interests and interdict others.^(7)^ Such phenomenon is explained by the way Merhy’s three suitcases are strategically arranged in a spectrum that defines the health care model. In models where hard technologies predominate, despite the effort to bring users into the world of technological action, medical knowledge does not fully overlap with what is intended by users; therefore, therapeutic projects are tense due to conflict established between the different wills of doctors and patients.^(7)^

Opposing soft and hard technologies may blur the potential relationships can have in technologically heavy environments such as ICUs, but as Merhy states, it is necessary to “promote exchanging procedure-centered physicians for care and relationship-centered ones ” and encourage the search for devices that allow autonomy of those who are cared for, using tensions as fuel for transformation.^(7)^

In such context, the concept of soft-hard technologies challenges the false opposition between soft and hard technologies and can place even intensive care in a field that is conducive to a conception of vulnerability that is instrumental for the transformation of practices, once these are established at a crossroads that is not definitely subjective, personal, singular or relational, like soft technologies, nor unrestrictedly material (or objective), like hard technologies. These technologies enable that which Ayres invites: the search for technological arrangements that are sensitive to individual and collective health needs in relational spaces that are supported by technology but extrapolate it, subverting it.^(8)^ In fact, in ICUs, medical knowledge, in all its theoretical-conceptual framework that encompasses different disciplinary fields, is vital to the care process, perhaps much more than the availability of hard technology, which is characteristic of soft-hard technologies. According to the author, when health is understood as a way of being, deciding to use technologies becomes an exercise of human autonomy that only applies to the moment of decision, translating the intersubjectivity of health care. The concept of vulnerability that we want to explore fits such description.

Ayres attributes three dimensions to vulnerability that allow for further broadening or narrowing of the concept and are identified by and related to perspectives and interests of subjects.^(5)^ These are the individual, social and programmatic dimensions of vulnerability, that guide the identification and articulation of explanatory elements to understand and respond to health problems.^(5)^ These dimensions also help understand the impact that expanding the concept of vulnerability might have on care in ICUs.

The individual dimension concerns the ability of each individual to experience illness and protect themselves from it, involving aspects of physical constitution and way of life.^(5)^ Without ignoring the importance of biological aspects, one can recognize the importance of the degree and quality of information that individuals have on their health status, their motivation and ability to understand and incorporate the information into their life practices.^(5)^ The social dimension is directly related to the contexts that shape individual vulnerabilities, for example, judicial-political structures, economic relations, gender relations, religious beliefs, poverty and social exclusion. All due to the fact that the ability to elaborate and incorporate information depends not only on individuals but also on access to communication, schooling, material resources and the power to influence political decisions and face cultural barriers.^(5)^ The programmatic dimension concerns the performance of institutions, especially those related to health, education, social welfare and culture, in reducing, reproducing or enhancing conditions of vulnerability.^(5)^

As Ayres has warned, by locating the target of interventions aimed at reducing vulnerability at the level of social susceptibility, even individual interventions extend beyond the simple task of warning, and thus need to overcome material, cultural and political obstacles that sustain vulnerability.^(5)^ According to the author, people need to know not only about health risks but also how to protect themselves and mobilize resources to transform structural situations that make them susceptible. In intensive care, hard technology is valued because it keeps people alive, but it does not change their way of life. Thus, the field of soft-hard technologies is especially conducive to interventions on vulnerability.

A mundane example can be seen when managing hypertensive emergencies in patients with decompensated systemic arterial hypertension in ICUs. Many patients labeled vulnerable by health professionals are those who reach serious disease states due to lack of access to effective services of lesser technological complexity or even due to lack of ability to understand and incorporate information about their own health. It is not uncommon for patients with systemic arterial hypertension to not adhere to treatment at asymptomatic stages, which is interpreted by many physicians as a personal choice based on their understanding of the disease, which in turn health professionals have difficulty changing, to improve adherence. These so-called nonadherent patients eventually suffer disease complications and reach emergency departments, being subsequently treated in ICUs. If an intensivist believes his or her role is only to reverse the organic condition and repeat medication guidelines for outpatient treatment, but that acting on vulnerability is out of scope because it is an inherent condition of those who *are* vulnerable, little is done in terms of tertiary prevention, when in fact, the scenario is also suitable to other care strategies. On the other hand, if an intensivist believes he or she has a preventive role in any environment, providing care is also an opportunity to transform the relationship one has with the disease itself, representing an opportunity to question which individual, social or programmatic elements interfere with the patients ability to incorporate treatment guidelines and develop overcoming strategies when appropriate, especially in light of a recent experience with severe illness, that can help sensitize patients about their own health status without necessarily blaming them.

### The potency of vulnerability

What about decisions that affect the health of those hospitalized in intensive care with whom it is not possible to establish intersubjective relationships? How powerful is the concept of vulnerability to solve such a dilemma, and how limited is it? Ayres points to a possible reply in his invitation to disrupt the idea that individuals are monads who act on the world only according to social imperatives, without the possibility of transforming reality.^(4)^ The author, therefore, urges us to avoid naturalizing vulnerability, taking it as an intrinsic characteristic of subjects.^(4)^ In fact, if, instead of understanding vulnerability as a fixed product of inequalities installed in certain populations, the choice is to use a relational concept of vulnerability and understand that the vulnerable are also modifying agents, other health practices become possible, as fluid as relationships are. It is not to say that communication, humanization and autonomy should lose ground in health care, but if they are judged from a purely ethical perspective, they will not be sufficient to guarantee the care that modern medicine prides on offering to meet collective and individual needs. It is necessary to incorporate broader and more powerful meanings of vulnerability, which challenge, including in intensive care settings, health care providers to see others as agents of transformation, whatever their condition may be.

Reflections on vulnerability presented herein are not intended to provide objective answers about what should or should not be decided. But rather, they seek to provoke questions about the care process that displace it, even if subtly, from the static position of doctor-caregiver and patient-care, to a place where relationships may become dialogic. Focusing on these issues might prove to be quite beneficial and fertile in intensive care settings.
